# Scoping review protocol on research prioritisation for preparedness and response to outbreaks of high consequence pathogens

**DOI:** 10.12688/openreseurope.15335.2

**Published:** 2024-04-29

**Authors:** Emilia Antonio, Dorothy Chepkirui, Shanthi Levanita, Susan Khader Ibrahim, Isabel Foster, Eli Harriss, Louise Sigfrid, Alice Norton

**Affiliations:** 1 Global Research Collaboration for Infectious Disease Preparedness (GloPID-R) Research and Policy Team, Global Research Collaboration for Infectious Pandemic Sciences Institute, University of Oxford, Oxford, UK; 2 Bodleian Health Care Libraries, University of Oxford, Oxford, OX3 9DU, UK

**Keywords:** High consequence pathogens, Preparedness, Response, Priority pathogens, Research prioritisation

## Abstract

**Background:**

Prioritisation of research activities for infectious disease pathogens is usually undertaken through the identification of important research and knowledge gaps. Research prioritisation is an essential element of both effective responses to disease outbreaks and adequate preparedness. There is however currently no published mapping of activities on and evidence from research prioritisation for high consequence pathogens. The objectives of this review are to map all published research prioritisation exercises on high-consequence pathogens; provide an overview of methodologies employed for prioritising research for these pathogens; describe monitoring and evaluation processes for research areas prioritised; and identify any standards and guidance for effectively undertaking research prioritisation activities for high consequence pathogens.

**Methods:**

The Joanna Briggs Institute guidance of scoping review conduct will be used. The search will be undertaken using the key terms of “research prioritisation”, “response”, “control”, and related terms, and a list of high-consequence pathogens derived from WHO (2020), EMERGE (2019), Europe CDC (2022) and the Association of Southeast Asian Nations (2021). We will search *WHO Global Index Medicus*; *Ovid Medline; Ovid Embase; Ovid Global Health; and Scopus*. Backward citations review of the included full text documents will also be conducted. *Google Scholar* and *Overton* will be searched for grey literature. Two independent reviewers will screen the retrieved documents using *Rayyan* and extract data in a data extraction template in Microsoft Excel 2021. Screening results will be presented using the PRISMA-ScR template with narrative synthesis undertaken for the extracted data.

**Conclusion:**

This review will map existing research priorities for high consequence pathogens. Further, it will provide an understanding of methodologies used for prioritisation, processes for monitoring and evaluation of progress made against research agendas, and evidence on standards that could be recommended for effective prioritisation of research for high consequence pathogens.

## Introduction

Prioritisation of health research is important for the allocation of limited resources to areas of greatest need. Understanding effective methodologies for prioritisation can help to inform guidance on best practice. Several approaches to setting research priorities for health are documented in the literature including consensus-based and metrics-based approaches which ultimately determine which priorities from many areas are put forward for further research focus ^1, 2^. Combinations of these two broad approaches are also utilised and, in some cases, an iterative process is adopted to narrow a list of priorities to the most essential ^3^.

In the response to outbreaks of new and re-emerging high consequence pathogens, with high morbidity and mortality, the need for evidence to contain infections and save lives is even more urgent, calling for rapid research prioritisation. For instance, multiple research priority setting activities were undertaken during the COVID-19 pandemic. These include the GloPID-R and WHO Global Research and Innovation Forum in January 2020 which yielded a Coordinated Global Research Roadmap for controlling the pandemic. Regionally-led activities such as those undertaken by the African Academy of Sciences (AAS) for outlining the COVID-19 research priorities for Africa and collaborative work undertaken by the Global Health Network (TGHN), UK Collaborative on Development Research (UKCDR) and the AAS for identifying research priorities for low- and middle-income countries (LMICs) are other examples ^4^.

A rapid start to vaccines R&D for COVID-19 led to the identification of several vaccine candidates within just over a year of the pandemic ^5^. Similarly, the prioritisation of vaccines research during the 2014 – 2016 West Africa Ebola outbreak resulted in the identification of an effective vaccine ^6^.

Preparedness priority setting involves anticipatory planning for future research needs in advance of disease outbreaks. The aim can be to prioritise the conduct of the relevant research prior to outbreaks and for research outputs to facilitate prevention and control of infections in the event of an outbreak. This “anticipatory” element could influence the selection of methods used in the identification of priorities to include those allowing for forecasting of uncertainties and consideration of mitigation measures to these ^7^. It is also important for priority setting activities for outbreak preparedness to take cognisance of research areas which might require the greatest research focus during an outbreak. These activities, undertaken in times of relatively less urgency, are likely to facilitate rapid responses when outbreaks occur.

There is consensus in the literature on the significant heterogeneity in approaches to developing and implementing health research priority agenda ^8, 9^. Variable methodologies and a lack of standardised reporting inhibits acceptability, credibility, and application of health research priorities. However, there is no agreement in the field on identified best method or group of methods for setting health research priorities ^10^. The most suitable methods are likely to be those which suit the purposes of the priority setting activities, are appropriate for the research context, and take cognisance of the available resources.

Several good practice principles for conducting and reporting on health research prioritisation exercises do exist and have been outlined in the literature including Cochrane guidance for prioritisation of systematic review topics, reporting guideline for priority setting of health research (REPRISE), and the COHRED research priority management guidance for national research priority setting ^11^. In a 2010 publication, Viergever *et al.* propose nine themes of good practice to be considered for priority setting for health research ^10^. This article describes, in addition to contextual considerations, inclusivity and systematic processes for planning and undertaking prioritisation, transparency, and effective communication as key elements for successful implementation of health research priorities identified ^10^.

WHO issued guidance for undertaking research priority setting activities for its staff in 2020. This drew from an assessment of WHO’s internal health research prioritisation activities across global, regional, and national contexts published between 2002 and 2017. The guidance is of broad scope, applicable in crises situations and in less urgent conditions, and is adaptable to the multi-levels of operations where WHO provides governance and technical support. As in other best practice documents on health research priority setting, planning for monitoring and evaluation processes at the time of identifying priorities are encouraged for enhancing effective implementation of research priority agenda ^8^.

### Rationale

Although broad guidance for undertaking health research prioritisation is available in the literature, there is limited discussion of priority setting guidance in the context of preparedness and response research for infectious disease outbreaks or any mapping of the existing research prioritisation landscape in this field. This scoping review of research prioritisation for preparedness and response to outbreaks of high consequence pathogens aims to address this gap.

The objectives of the review are:

1. To map published research prioritisation exercises for high consequence pathogens.
2. To provide a descriptive analysis of methodologies used in priority setting for preparedness and response research for high consequence pathogens.
3. To describe assessment processes for monitoring progress on prioritised research areas.
4. To identify any standards or guidance for effectively undertaking research prioritisation activities for high consequence pathogens.

The idea to undertake this review emerged as a result of work undertaken with the GloPID-R LMICs working group. This group of research funders, which has been undertaking consultations and discussions amongst its funder members and researchers on research prioritisation for disease during the current COVID-19 pandemic, has identified best practice considerations for priority setting for disease outbreaks from this experience.

There are several definitions of high consequence pathogens and various international, regional, and national organisations have shortlisted priority pathogens based on parameters which are often unclear. Whilst some priority pathogen lists focus on viral pathogens only, others include antimicrobial resistant pathogens and classifications based on pathogens for which no effective countermeasures exist ^12, 13^.

Noting this variability, for the purposes of this scoping review, we define high consequence pathogens as infectious disease pathogens which cause diseases in humans with the potential to cause outbreaks associated with devastating morbidity and mortality. We will include in this review priority pathogens lists identified by the WHO and regional public health organisations (where publicly available) as shown in Table 1. We did not identify any regional priority pathogen lists for the Africa region and the Americas at the time this review was initiated. To be inclusive in the scope we have also included broad search terms on research prioritisation to capture any wider activities in the published and grey literature not specifically linked to any of the listed pathogens included.

**Table 1.** High consequence pathogens to be included in the scoping review and corresponding source organisations by which they were identified.

Pathogens	Global	Europe		Asia-Pacific
	WHO	EMERGE	ECDC	ASEAN
COVID-19 (SARS-CoV2)	X		X	X
Crimean-Congo haemorrhagic fever virus	X	X		
Ebola virus	X	X		
Marburg virus disease	X			
Lassa fever virus	X	X		
Middle East respiratory syndrome coronavirus	X			
Severe Acute Respiratory Syndrome (SARS)	X			
Nipah and henipaviral diseases	X	X		X
Rift Valley fever	X			
ZikaVirus	X			
“Disease X”	X			
Smallpox		X		
Hendra Virus		X		
Swine Influenza A (H3N2)			X	
Highly pathogenic avian influenza A (H5N1)				X
Low pathogenic avian influenza H7N9				X
Pandemic influenza A (H1N1) 2009 virus				X
Polio virus			X	
Monkey Pox			X	
West Nile Virus			X	
Brucellosis ( *Brucella bacteria*)		X		
Q Fever ( *coxiella burneti*)		X		
Melioidosis ( *Burkholderia pseudomallei*)		X		
Tularaemia ( *Francisella tularensis*)		X		
Cholera ( *Vibrio cholera*)		X		
Plague ( *Yersinia pestis*)		X		
Glanders ( *Burkholderia mallei*)		X		
Anthrax ( *Bacillus anthracis*)		X		

## Methods

To our knowledge, there is no existing systematic mapping for research prioritisation activities for preparedness and response to high consequence pathogens. The review will be undertaken in accordance with the Joana Briggs Institute methodology for scoping reviews.

Initially, we reviewed a list of key resources which were compiled as part of the activities of the GloPID-R Research in LMICs working group. These resources were reviewed and used to develop a list of search terms for the search strategy based on the objectives of the review. Eligibility criteria were defined including: (a) articles focused on research prioritisation in preparedness for and in response to outbreaks of high consequence pathogens and assessment strategies; (b) peer reviewed articles, policy papers, reports, commentaries, or roadmaps of global, regional, or national scope; and (c) the selected list of high consequence pathogens ( Table 1). There will be no limits for year of publication or language unless the articles cannot be translated using *Google Translate*. Literature on antimicrobial resistance is out of the scope of this review apart from where it explicitly relates to one of the pathogens selected.

### Search strategy

The search strategy will aim to identify both peer reviewed and grey literature. Using the search terms identified from the compiled resources, an initial search will be done on *Ovid Embase.* The indexed terms identified will be used to build a final strategy, with adaptations to be made for the other databases. The following databases will be searched for this scoping review: *WHO Global Index Medicus*; *Ovid Medline*; *Ovid Global Health*; and *Scopus.* A review of bibliographies of the articles included and extracted from these databases will be conducted. A review of backward citations of included full texts will be done. Sources of unpublished grey literature will include *Google Scholar* and *Overton*. The proposed search strategy is provided as extended data ^14^.

### Study/source of evidence selection

Following the search, all identified citations will be uploaded to EndNote 20 and duplicates removed using the Deduklick programme developed by Risklick. Zotero could also be used to perform a similar function to EndNote, and R-Accelerator Deduplicator programme as an alternative to Deduklick. These will then be exported to Rayyan for initial title and abstract screening against the inclusion and exclusion criteria. Potentially relevant articles will be selected, and full text screening done. The reasons for exclusion at this stage will be recorded and reported in the scoping review. Screening will be blinded on Rayyan to be done independently by two reviewers with conflicts settled by a third independent reviewer. The results of the search and screening will be presented in Preferred Reporting Items for Systematic Reviews and Meta-analyses extension for scoping review (PRISMA-ScR) flow diagram ^15^.

### Data extraction

Data will be extracted from articles included in the scoping review by two independent reviewers using a data extraction table developed a priori. The data extracted will include specific details about the pathogen of interest, scope of the article, prioritisation methodologies used, monitoring and evaluation procedures and recommendations for best practice (see Table 2). A pilot of the data extraction tool will be done using the first five articles already collated as key resources for this review. This will be used to refine the tool before extraction by the two reviewers.

**Table 2.** Data extraction table.

Field	Description	Details
1. Map research prioritisation activities		
**Title**	Title of the article	
**Year of publication**	Publication year as documented	
**Type of article**	Article type as documented	E.g., policy paper, journal article, reports
**Author**	All named authors documented	All named authors documented
**Author affiliation**	Institutional affiliation of author(s)/ Organisation leading prioritisation activities	
**Version of research agenda**	Version of research agenda as documented	Version 1, version 2 etc.
**Duration of research agenda**	Duration of validity of research priorities	E.g. strategy for 1 year, 6 months etc)
**Pathogen(s) of interest**	Indicate the pathogen(s) that are addressed in the article	E.g., COVID-19, coronaviruses, Ebola etc.
**Purpose**	The main purpose of the article e.g., preparedness or response	preparedness, response, or both
**Key focus**	Key discipline area of focus	e.g. vaccine research for COVID-19, mental health research for Ebola
**Geographical target**	Country/ies or region(s) to which the priority setting is designed for	national, region(s), global, others
		If "other" state level
		Details of countries and regions
**Target population**	Indicate the populations targeted where available	e.g., children, women, pregnant women, indigenous populations etc
2. Identify priority setting methodologies		
Named methodology employed	Indicate name of method where available	e.g., CAM 3D, CHNRI, COHRED etc
Type of methodology employed	Type of method employed for prioritisation	Delphi survey, surveys, nominal group technique, interviews, focus groups, meetings, workshops, literature review/ gap analysis, Other etc.
		if "other" describe
Brief description of methodology	Describe method used briefly	
Duration of priority setting process	Duration in days, months, years as documented	
Criteria used to group the priorities	Criteria described in grouping priorities	E.g., emergent, pre-determined
Method used to group the priorities	Methods described in grouping priorities	E.g., voting, ranking; etc
Criteria for shortlisting priorities	Criteria used for selecting priorities as documented	E.g., need, feasibility, novelty, equity, timing (immediate/long-term)
Mode of consultation	Mode of consultation as described	E.g., In-person, online
Stakeholders/ contributors	Participants involved in the priority setting if there were deliberations/surveys/meetings etc., include the countries of contributors	E.g., patients, clinicians, public/ communities, policymakers, funders, experts/ researchers
Communication plan	An outline of strategies to be used in disseminating the set priorities	E.g., publications, press release, website launch etc
3. Describe monitoring and evaluation (M&E) activities		
Processes for M&E	An outline of key M&E activities planned/undertaken for the set priorities	
Review period	Indicate planned timeline for review	E.g., annual or 5-year period
Responsible entities	Who is responsible for the M&E and any regulations for that/any plans for M&E	
4. Identify best practice recommendations		
Recommendations	An outline of recommendations for future priority setting, for response or outbreak preparedness	
Guidance for priority setting	Indicate standards/guidance recommended as listed	E.g., WHO guidance for priority setting, CHNRI

Acronyms - CAM 3D: Combined Approach Matrix 3D, CHNRI: Child Health and Nutrition Research Initiative, COHRED: Council on Health Research for Development

The draft data extraction tool will be modified and revised as necessary during the process of extracting data from each included evidence source. Modifications made will be explained and justified in the scoping review. Two independent reviewers will undertake data extraction and any arising disagreements will be resolved through discussion with the overall scoping review lead.

### Data analysis and presentation

Data will be analysed thematically in Microsoft Excel 2021 by examining broad themes under each section of data extracted and guided by the objectives of the scoping review. Tabular and graphical presentations accompanied by narrative summaries will be used to provide a mapping of the different methodologies for priority setting for high consequence pathogens, monitoring and evaluation processes, and recommendations/guidance for best practice. Findings will be disseminated in a peer reviewed publication and to key stakeholders in the field including funding organisations, researchers, and policy stakeholders.

### Study status

An initial search for the published literature has been conducted using the proposed search strategy. The search is expected to be completed by December 2022 and final analysis completed as of mid-2023.

## Ethics and consent

Ethical approval and consent were not required.

## Data Availability

There is no underlying data associated with this article. figshare: Scoping Review Protocol: Search Strategy. https://doi.org/10.6084/m9.figshare.21739544 ^14^ Data are available under the terms of the Creative Commons Attribution 4.0 International license (CC-BY 4.0).
