# Supplementary material for: Scoping review protocol on research prioritisation for preparedness and response to outbreaks of high consequence pathogens
Source: Open Res Eur. 2024 Apr 29;3:16. Originally published 2023 Jan 25. [Version 2] doi: 10.12688/openreseurope.15335.2 (PMC10445874; doi:10.12688/openreseurope.15335.2)
Supplement: Peer review history

# Reviewer report: Reviewer response for version 1

**DOI:** 10.21956/openreseurope.16578.r33933

**Published:** 2023-08-25

**Authors:** Mark K Huntington

**Affiliations:** 1 University of South Dakota Sanford School of Medicine, Sioux Falls, USA

**OVERVIEW**

 This manuscript presents the methodology for review of the literature on establishing research priorities for infectious outbreaks. The recent pandemic emphasized the importance of establishing such a rational and prioritized strategy for meaningful investigations of emerging disease – such a strategy is not apparent in the significant bulk of COVID work published to date.

 The method of the proposed study is well-designed and well-described.  The subsequent project of collating previous published reports on the topic has great potential to make a meaningful contribution to the literature once it is completed.

**COMMENTS AND SUGGESTIONS** (by page.column.paragraph of the PDF version)

- 3.2.2 Citation 11 refers to REPRISE; please include citations for Cochrane and COHRED as well.
- 4 (Table 1) The table is incomplete. It is notable for its lack of inclusion of pathogen lists for organizations from the Americas or Africa. Surely such organizations and lists exist and they should be included in columns along with Europe and Asia-Pacific organizations, given the global significance of this study (or else please add a convincing reason for their exclusion). 
- 5.2.2 Please include citations for the tools mentioned in this paragraph.
- 7.2 Reference 9 does not include complete bibliographic details. Please add them.

Is the study design appropriate for the research question?

Yes

Is the rationale for, and objectives of, the study clearly described?

Yes

Are sufficient details of the methods provided to allow replication by others?

Yes

Are the datasets clearly presented in a useable and accessible format?

Not applicable

Reviewer Expertise:

Primary Care/General Medicine, Disaster Medicine, Global Health, Microbiology, Medical Education

I confirm that I have read this submission and believe that I have an appropriate level of expertise to confirm that it is of an acceptable scientific standard.

# Reviewer report: Reviewer response for version 1

**DOI:** 10.21956/openreseurope.16578.r33934

**Published:** 2023-07-24

**Authors:** Pasi Penttinen

**Affiliations:** 1 School of Transnational Governance, European University Institute, Fiesole, Toscana, Italy

This is a clearly described and appropriately documented protocol to study a relevant operational question on mapping research priority exercises and methods for high-risk pathogens. The study has relevance for national and international research funders and public health authorities.

 I have only two minor concerns:

1. Will the proposed approach will capture adequately other languages than English? I would have assumed that the search terms need to be translated to relevant major languages for that to work.
2. I would consider for grey literature also searching specifically on the websites of 20 major research funders and public health authorities.

Is the study design appropriate for the research question?

Yes

Is the rationale for, and objectives of, the study clearly described?

Yes

Are sufficient details of the methods provided to allow replication by others?

Yes

Are the datasets clearly presented in a useable and accessible format?

Yes

Reviewer Expertise:

Public health, respiratory viruses, epidemic intelligence

I confirm that I have read this submission and believe that I have an appropriate level of expertise to confirm that it is of an acceptable scientific standard.

# Response

**Published:** 2023-08-25

**Authors:** Emilia Antonio

**Affiliations:** Global Research Collaboration for Infectious Diseases Preparedness, UK

Thank you for your suggestions for improving the scoping review. We recognised the challenges with identifying non-English publications and addressed these in our search approach by:

1. Including bibliographic databases which ensured that a range of non-English journals were already included. For example, see Content Coverage Guide for Scopus. Further, records indexed in the WHO Global Index Medicus database have English language translations of the journal article titles as well as the original title. 
2. Including thesaurus terms or controlled vocabulary terms, where relevant, when searching the bibliographic databases to retrieve records (including non-English records) if they have the relevant thesaurus terms in that field in the database.

We look forward to sharing the results of the review in due course.

# Reviewer report: Reviewer response for version 1

**DOI:** 10.21956/openreseurope.16578.r30733

**Published:** 2023-02-01

**Authors:** Robert Terry

**Affiliations:** 1 TDR - The Special Programme for Research and Training in Tropical Diseases, World Health Organization, Geneva, Switzerland

The protocol is clear. Perhaps clarify the reference to WHO will also include outputs from the 6 WHO Regional Offices - these regional publications might only be available via the regional websites. The WHO publication repository, IRIS, might need to be searched specifically as publications in there might not appear in your other search strategies, see https://apps.who.int/iris/.

 Objective 1 & 2 are straightforward. Very good to see objective 3. The new WHO guidance has a section on how to measure awareness, uptake, progress change in funding patterns, change in funding volumes etc. which you might want to use to categorise 'progress' .

 I think 4 is a challenge but good to see it there. 

 As each reference that is within scope will be read in full I would recommend that the *purpose *of the research is classified to give an overview of priorities identified across the full scope of research. The framework of 5 areas - set out in reference 8 - would be one approach to consider. By doing this you would obtain a high-level heat map of the priorities you have identified and potentially areas that are not prioritised. It will also provide a quick easy to understand visual to summarise your work.

Is the study design appropriate for the research question?

Yes

Is the rationale for, and objectives of, the study clearly described?

Yes

Are sufficient details of the methods provided to allow replication by others?

Yes

Are the datasets clearly presented in a useable and accessible format?

Yes

Reviewer Expertise:

Research policy, priority setting, open science, evidence informed policy.

I confirm that I have read this submission and believe that I have an appropriate level of expertise to confirm that it is of an acceptable scientific standard.

# Response

**Published:** 2023-02-22

**Authors:** Emilia Antonio

**Affiliations:** Global Research Collaboration for Infectious Diseases Preparedness, UK

Thank you for your very useful comments and suggested improvements for the scoping review. These will certainly enrich the review. We look forward to sharing our findings in due course.
